# The Silent Surge: Obesity Driving a Global Cardiovascular Crisis

**DOI:** 10.5334/gh.1464

**Published:** 2025-09-03

**Authors:** Panniyammakal Jeemon, Sivasankaran Sivasubramonian

**Affiliations:** 1 Sree Chitra Tirunal Institute for Medical Sciences and Technology, Trivandrum, Kerala, India; 2 Manipal Academy of Higher Education, Manipal, Karnataka, India

**Keywords:** Obesity, Cardiovascular disease, Roadmap, Interventions, Equity

## Abstract

Recent global estimates indicate that more than one billion people live with obesity, a figure that has doubled since 1990. When overweight individuals are included, nearly 2.5 billion adults are affected, with high body mass index contributing to an estimated 1.9 million cardiovascular disease (CVD) deaths annually.

Obesity and its close association with CVD remain pressing public health challenges that require sustained, coordinated action. Recent global policy discussions, including the UN General Assembly’s Zero Draft Political Declaration, highlight the importance of improving food labelling, taxing sugary drinks, limiting the marketing of unhealthy foods, and encouraging active living through supportive urban planning. Countries are encouraged to align national obesity strategies with the WHO’s 2022 Acceleration Plan to STOP Obesity, with clear goals and mechanisms for accountability and monitoring.

Preventive measures are most effective when introduced early, such as encouraging breastfeeding and creating supportive school environments that offer balanced meals, limit access to unhealthy foods, and incorporate regular physical activity into daily schedules. Fiscal measures, including taxes, subsidies, and mandatory nutrition labels, can help guide consumer choices towards healthier options. Supportive built environments with safe access to parks, pedestrian routes, and cycling paths further encourage active lifestyles.

Health systems are central in ensuring equitable access to prevention and treatment, delivered through stigma-free and evidence-based care. Community-based and family-oriented programs have shown promise, while pharmacological options may complement lifestyle approaches where appropriate. Long-term progress depends on sustained commitment, cross-sectoral collaboration, and integration of obesity prevention into broader public health frameworks.

## Obesity burden

Obesity is a complex, long-term disease condition influenced by biological, environmental, and social factors, and it stands as one of the most significant public health issues of our time. In a recent publication in *Global Heart*, Lopez-Jimenez and colleagues presented an in-depth analysis of international data, revealing that over one billion people around the world are currently living with obesity, as defined by a high body mass index (BMI), a figure that has doubled since 1990 (1). When including those who are overweight, the global total climbs to nearly 2.5 billion adults affected by excess weight. The study also estimated that approximately 1.9 million cardiovascular disease (CVD) deaths each year are directly tied to high BMI, underscoring the widespread impact of the condition. Over the last 30 years, the age-adjusted rates of obesity and obesity-related CVD mortality have both doubled, emphasising the pressing need for comprehensive and coordinated policy responses (1). Despite these serious health consequences and premature mortality, obesity continues to receive limited attention in global health strategies and planning. The work by Lopez-Jimenez et al. represents a significant contribution, providing robust data to guide policy responses to this escalating crisis.

## Why this matters: Social, economic, and health system impacts

The pandemic of obesity is no longer confined to high-income nations. It has become universal, with rapid and disproportionate increase observed in low- and middle-income countries (LMIC) and an alarming shift in global health patterns (1). For individuals, high BMI elevates the lifetime risk of heart attack, stroke, and other cardiovascular events, sometimes by two to three times compared to people with a healthy weight. CVD attributable to obesity reduces life expectancy, increases morbidity, and impairs quality of life (2). At the health system and societal level, obesity and associated CVD create unsustainable fiscal pressures. Estimates indicate that, without effective prevention and treatment, the economic impact of obesity could reach USD 4.3 trillion annually by 2035, equal to nearly 3% of global GDP (3). The excess CVD burden strains health infrastructure, elevates healthcare costs, and erodes economic productivity. The consequences are potentially catastrophic in LMIC, where systems are even more fragile.

While it is essential to document and quantify the scale of the obesity crisis accurately, such efforts alone are insufficient to halt its relentless progression. Merely cataloguing the statistics and health consequences does not translate into meaningful change unless decisive, coordinated policy actions at the global level accompany it. Obesity is driven by a complex mix of biological, social, economic, and environmental factors, demanding a comprehensive and sustained response. Effective strategies must go beyond awareness to target root causes, such as unhealthy food systems, sedentary behaviour, and unequal access to healthcare, through prevention, better management, and supportive environments. Global collaboration is essential to align efforts, pool resources, and ensure accountability across sectors. Without strong political will and a clear, actionable roadmap, obesity will continue to strain healthcare systems and deepen health inequalities. The time has come to shift from observation to implementation, using evidence-based policies to reduce obesity rates and improve health outcomes worldwide.

## A roadmap for global prevention: Policy must lead the way

Addressing the obesity–cardiovascular disease crisis requires long-term, coordinated action across sectors, involving governments, public health agencies, and international bodies. The UN General Assembly’s Zero Draft Political Declaration recognises obesity as a major public health issue and a leading cause of CVD (4). It calls for urgent, wide-ranging measures, including restricting unhealthy food marketing, taxing sugary drinks, improving food labelling, and promoting physical activity through better urban and transport planning. The declaration also emphasises equitable access to prevention, early diagnosis, and treatment of obesity, while strengthening health systems. It urges global cooperation, resource mobilisation, and monitoring systems to drive and track progress effectively.

**Figure 1:**
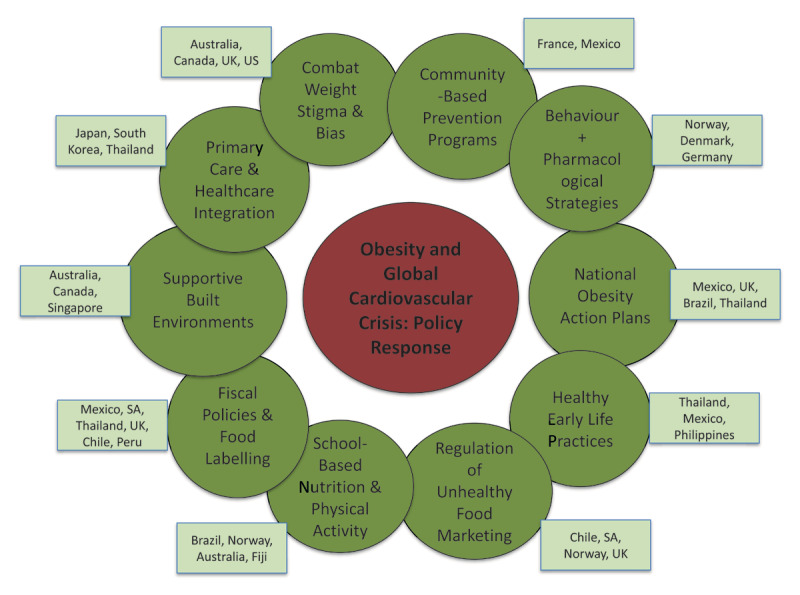
Policy response to the global cardiovascular crisis arising from obesity. UK = United Kingdom, SA = South Africa, US = United States of America.

Evidence-based strategies can provide a roadmap for effective intervention in obesity risk reduction (Figure 1). Countries are encouraged to adopt and implement national obesity action plans aligned with the WHO’s 2022 Acceleration Plan to STOP Obesity (5), with clearly defined targets through 2030. These plans should involve coordinated efforts across health, education, agriculture, urban planning, finance, and transport sectors, supported by accountability, financing, and surveillance mechanisms.

Promoting healthy practices from early life is essential, including the protection and promotion of breastfeeding, which has long-term benefits for healthy weight and cardiovascular risk reduction. Restricting the marketing of unhealthy foods and sugar-sweetened beverages, particularly in environments frequented by children, can help reduce consumption of calorie-dense, nutrient-poor products. In schools, nutrition policies should prohibit high-fat, sugar, and salt foods near campuses, ensure healthy cafeteria offerings, and embed physical activity standards into curricula to encourage lifelong healthy habits.

Fiscal measures such as taxing sugary drinks, providing incentives for healthy foods, and introducing mandatory, clear nutritional labelling can guide consumer choices towards more nutritious diets. Built environments could encourage active living by ensuring safe access to recreational spaces, parks, pedestrian routes, and cycling infrastructure. In the healthcare system, obesity prevention and treatment may be integrated into primary care, with equitable access to multidisciplinary programs delivered by providers trained to offer stigma-free, evidence-based care.

Addressing weight stigma and bias in healthcare and society is necessary to ensure fair treatment and foster patient engagement. Community-based prevention programs, particularly those targeting children, can be highly effective; for example, the EPODE model achieved a 50% relative reduction in overweight prevalence in participating towns compared to the comparison towns (6). Family-based structured lifestyle modification interventions are promising in achieving population-average weight reduction (7) and are cost-effective (8) even in LMIC settings. Finally, while lifestyle modification remains the foundation of obesity management, newer pharmacological treatments can complement these efforts, provided cost, accessibility, and long-term sustainability are considered. A concerted and equitable approach combining prevention, lifestyle interventions, and pharmacological management may offer the best hope for reducing the burden of obesity worldwide.

Whether through food labelling, taxation, education, or built environment changes, countries that invest in structured, government-led plans, grounded in prevention and equity, have seen real progress in shifting behaviours and, in some cases, stabilising or reducing obesity prevalence (Table 1). Taken together, these measures require strong coordination, sustained commitment, and integration into wider public health frameworks to halt the rise in obesity and reduce the burden of cardiovascular disease.

**Table 1:** Elements in National Obesity Reduction Efforts.


COUNTRY	KEY POLICIES	IMPACT

**Chile (16)**	Front-of-pack warning labels, marketing restrictions, junk food bans in schools.	Drop in sugary drink purchases; decrease in sodium content and saturated fat; improved nutrition understanding; industry reformulation.

**UK (17)**	Sugar tax, calorie targets, and food labelling.	Sugar reduction in soft drinks and less sugary drink intake.

**Mexico (18)**	Sugar/food taxes and marketing limits.	Drop in sugary drink purchases; more substantial effect in low-income groups; improved diets.

**France (6)**	ÉPODE program via schools, parents, and local leaders.	Obesity reduction in pilot towns; replicated in other countries.

**South Korea (19)**	National health plans, food labelling.	Stable child obesity; higher awareness of healthy diets.

**Australia (20)**	Health Star Rating, Healthy Food Partnership, and school initiatives.	Labelling improved awareness; substantial population health gain.

**Brazil (21)**	Traditional diets, dietary guidelines, and school meals from local farms.	High trust in guidelines; healthier school meals; cultural food habits preserved

**South Africa (22)**	Sugar tax, dietary guidelines, school nutrition programs.	Drop in sugary drink intake; product reformulation; strong impact on low-income groups.

**Thailand (23)**	Front-of-pack labelling, school reforms, ThaiHealth campaigns.	Reduced sugary drink intake; industry reformulation; community health engagement.

**Peru (23)**	Front-of-pack black octagons, school food/ad bans.	Better consumer understanding, improved purchasing habits, and increased industry compliance.


## Response from the Global South

Countries in the Global South are addressing obesity through a combination of fiscal policies like sugar taxes (seen in Mexico, South Africa, and Thailand), reforms in food environments such as front-of-pack labelling and healthier school food standards, and public awareness campaigns, including India’s Fit India and Brazil’s food guidelines (Table 1). These efforts often involve community engagement to support traditional diets, integration of obesity prevention into health systems (as in Thailand and Fiji), and alignment with WHO strategies. Despite limited resources, progress is evident where there is strong political commitment, regulatory action, and long-term equity-focused planning, demonstrating that multifaceted, locally tailored approaches can effectively reduce obesity and related cardiovascular risks (9).

## Role of behavioural economic nudges in obesity prevention and control

Nudges are small environmental changes that encourage healthier choices without restricting individual freedom. Based on behavioural insights such as defaults, social norms, and framing effects, nudges influence subtly but measurable dietary and physical activity behaviours and show considerable potential in reducing obesity risk (Table 2). When thoughtfully designed and integrated into comprehensive public health policies, these approaches can help shape environments supporting sustained, population-wide diet and physical activity improvements.

Default options make use of the tendency to accept pre-set choices. Making healthier items the default option in food settings can improve nutrition without coercion; for example, in university canteens, nudging improved sustainable food consumption patterns (10).

**Table 2:** Global Evidence of Behavioural Economic Nudges in Obesity Reduction.


NUDGE TYPE	DESCRIPTION	EVIDENCE OF IMPACT

**Default Options**	Pre-setting healthier choices as defaults.	Fruit is offered as the default. Fruit consumption increased.

**Product Placement**	Positioning healthy foods prominently.	Increased sales of fruits and vegetables; higher selection of water over sugary drinks.

**Social Norms Messaging**	Promoting healthy behaviours as standard/socially accepted.	Increased stair usage; increased fruit and vegetable consumption.

**Portion Size Reduction**	Serving smaller portions or using smaller plates.	Reduced calorie intake without compensatory eating.

**Framing Effects**	Presenting nutritional information in positive/negative frames.	Positive framing improved healthy food choices; calorie labels reduced caloric intake.

**Incentives & Rewards**	Financial or voucher-based rewards for healthy behaviour.	Increased purchase of healthy foods; improved physical activity adherence.

**Commitment Devices**	Encouraging public commitments to health goals.	Improved weight loss maintenance and diet adherence.


This summary table underscores the diversity and efficacy of behavioural economic nudges tested worldwide. While effects vary by context and design, these nudges are valuable, low-cost tools that can complement broader obesity prevention strategies globally.

Product placement and visibility are also powerful nudging techniques. Positioning healthier foods at eye level or in prominent locations increases the likelihood of their selection. In the United Kingdom, supermarkets involved in the Public Health Responsibility Deal boosted fruit and vegetable sales by placing them in more visible areas (11). Schools have applied similar strategies by reorganising cafeterias to make water and healthy snacks more accessible than sugary drinks and processed foods, encouraging better dietary choices among children (12).

Monetary incentives and pricing strategies also draw on behavioural economics. Taxes on sugary drinks discourage consumption, while small rewards for healthy choices reinforce positive habits. In Singapore, a pilot program offering grocery vouchers to families who bought fruits and vegetables successfully increased their intake (13).

Nudges are generally low-cost, scalable, and respectful of individual autonomy, making them appealing for public health interventions. However, they are most effective when part of broader strategies that address structural barriers such as limited food access, economic inequality, and cultural preferences (14). Ethical considerations are also important; interventions must be transparent and uphold personal freedom.

## Equity must anchor the response

A truly effective global response must be grounded in equity. Low- and middle-income countries face the most rapid increases in obesity and related cardiovascular disease, yet often lack the resources and infrastructure needed to respond effectively. Support through financial aid, technology transfer, and international collaboration is essential. Policies must tackle the underlying social determinants of health, as obesity and cardiovascular disease are shaped by gender, socio-economic inequalities, and the coexistence of undernutrition and overnutrition. Interventions should be culturally sensitive, inclusive, and designed to reach marginalised populations who bear a disproportionate share of the risk. Achieving meaningful progress ultimately depends on political will. Governments and global institutions must acknowledge obesity as a chronic disease and respond with the sustained commitment that such recognition demands (15).

## References

[1] Lopez-Jimenez F, Di Cesare M, Powis J, Shrikhande S, Adeoye M, Codato E, Zhou B, Bixby H, Evans N, Lara-Breitinger K, Rodriguez MA, Hadeed L, Barquera S, Taylor S, Perel P, Pineiro D, Narula J, Pinto F. The Weight of Cardiovascular Diseases: Addressing the Global Cardiovascular Crisis Associated with Obesity. Glob Heart. 2025 Aug 21;20(1):68. DOI: 10.5334/gh.1451. PMID: 40860963. PMCID: PMC12372701. 40860963 PMC12372701

[2] Chen Q, Huang S, Wang X, Peng J, Wang P, Luo R, Shi X, Xu H, Zhang W, Shi L, Peng Y, Wang N, Tang X. The burden of diseases attributable to high body mass index in Asia from 1990 - 2019: results from the global burden of disease study 2019. Ann Med. 2025 Dec;57(1):2483977. DOI: 10.1080/07853890.2025.2483977. Epub 2025 Mar 28. PMID: 40151071. PMCID: PMC11956100. 40151071 PMC11956100

[3] World Obesity Federation. World Obesity Atlas 2023. London, UK: WOF; 2023.

[4] World Health Organization. Zero draft: political declaration of the fourth High-Level Meeting of the General Assembly on the prevention and control of noncommunicable diseases and the promotion of mental health and well-being. Geneva, Switzerland: WHO; 2025.

[5] World Health Organization. The WHO Acceleration Plan to STOP Obesity: progress from WHA 75 Nutrition and Food Safety Department. Geneva, Switzerland: WHO; 2023.

[6] Borys JM, Valdeyron L, Levy E, Vinck J, Edell D, Walter L, et al. EPODE – a model for reducing the incidence of obesity and weight-related comorbidities. Eur Endocrinol. 2013;9(2):116–120. DOI: 10.17925/USE.2013.09.01.32 29922365 PMC6003578

[7] Panniyammakal J, Stanley A, Ismail S, Lekha TR, Ganapathi S, Harikrishnan S. Family-based interventions to promote weight management in adults: results from a cluster randomized controlled trial in India. Ann Fam Med. 2025;23(2):93–99. DOI: 10.1370/afm.230632 40127979 PMC11936353

[8] John AS, Ganapathi S, Harikrishnan S, Lekha TR, Stanley A, Soman B, et al. Within-trial cost-effectiveness analysis of a family-based structured lifestyle modification intervention program for cardiovascular risk reduction: results from the PROLIFIC trial. Glob Heart. 2025;20(1):65. DOI: 10.5334/gh.1450 40757252 PMC12315683

[9] NCD Risk Factor Collaboration (NCD-RisC). Worldwide trends in underweight and obesity from 1990 to 2022: a pooled analysis of 3663 population-representative studies with 222 million children, adolescents, and adults. Lancet. 2024 Mar 16;403(10431):1027–1050. DOI: 10.1016/S0140-6736(23)02750-2. Epub 2024 Feb 29. PMID: 38432237. PMCID: PMC7615769. 38432237 PMC7615769

[10] Torris C, Mobekk H. Improving cardiovascular health through nudging healthier food choices: a systematic review. Nutrients. 2019;11(10):2520. DOI: 10.3390/nu11102520 31635377 PMC6836015

[11] Baird J, Dhuria P, Payne H, Crozier S, Lawrence W, Vogel C. Implementation of a UK supermarket intervention to increase purchasing of fresh fruit and vegetables: process evaluation of the WRAPPED natural experiment. Int J Behav Nutr Phys Act. 2024;21(1):128. DOI: 10.1186/s12966-024-01679-3 39529139 PMC11552182

[12] https://iris.who.int/bitstream/handle/10665/107797/E89501.pdf?sequence=1&isAllowed.

[13] https://iris.who.int/bitstream/handle/10665/260253/WHO-NMH-PND-16.5Rev.1-eng.pdf.

[14] Harbers MC, Beulens JWJ, Rutters F, de Boer F, Gillebaart M, Sluijs I, van der Schouw YT. The effects of nudges on purchases, food choice, and energy intake or content of purchases in real-life food purchasing environments: a systematic review and evidence synthesis. Nutr J. 2020 Sep 17;19(1):103. DOI: 10.1186/s12937-020-00623-y. PMID: 32943071. PMCID: PMC7500553. 32943071 PMC7500553

[15] Luli M, Yeo G, Farrell E, Ogden J, Parretti H, Frew E, Bevan S, Brown A, Logue J, Menon V, Isack N, Lean M, McEwan C, Gately P, Williams S, Astbury N, Bryant M, Clare K, Dimitriadis GK, Finlayson G, Heslehurst N, Johnson B, Le Brocq S, Roberts A, McGinley P, Mueller J, O’Kane M, Batterham RL, Miras AD. The implications of defining obesity as a disease: a report from the Association for the Study of Obesity 2021 annual conference. EClinicalMedicine. 2023 Apr 6;58:101962. DOI: 10.1016/j.eclinm.2023.101962. PMID: 37090435. PMCID: PMC10119881. 37090435 PMC10119881

[16] Taillie LS, Bercholz M, Popkin B, Reyes M, Colchero MA, Corvalan C. Changes in food purchases after the Chilean policies on food labelling, marketing, and sales in schools: a before and after study. Lancet Planet Health. 2021;5(8):e526–e533. DOI: 10.1016/S2542-5196(21)00172-8 34390670 PMC8364623

[17] Luick M, Bandy LK, Harrington R, Vijayan J, Adams J, Cummins S, et al. The impact of the UK soft drink industry levy on the soft drink marketplace, 2017–2020: an interrupted time series analysis with comparator series. PLoS One. 2024;19(6):e0301890. DOI: 10.1371/journal.pone.0301890 38843248 PMC11156274

[18] Colchero MA, Popkin BM, Rivera JA, Ng SW. Beverage purchases from stores in Mexico under the excise tax on sugar sweetened beverages: observational study. BMJ. 2016;352:h6704. DOI: 10.1136/bmj.h6704 26738745 PMC4986313

[19] World Health Organization. South Korea: Front-of-Pack Labeling (FOPL) Policy. Global database on the Implementation of Food-related Nutrition Action (GIFNA). Geneva, Switzerland: WHO; 2022.

[20] Cleghorn CL, Blakely T, Bablani L, Ni Mhurchu C. Estimated health impacts of reformulation resulting from Health Star Rating nutrition labelling in Aotearoa New Zealand. J R Soc N Z. 2025;55(6):1904–1920. DOI: 10.1080/03036758.2025.2455499 40756821 PMC12315154

[21] Food and Agriculture Organization of the United Nations. School feeding programs in brazil: supporting sustainable diets and food systems. Rome, Italy: FAO; 2019.

[22] Kruger HS, van Zyl T, Monyeki MA, Ricci C, Kruger R. Decreased frequency of sugar-sweetened beverages intake among young children following the implementation of the health promotion levy in South Africa. Public Health Nutr. 2025;28(1):e23. DOI: 10.1017/S1368980024002623 39764638 PMC11822614

[23] Afroza U, Abrar AK, Nowar A, Sobhan SMM, Ide N, Choudhury SR. Global overview of government-endorsed nutrition labeling policies of packaged foods: a document review. Front Public Health. 2024;12:1426639. DOI: 10.3389/fpubh.2024.1426639 39583081 PMC11581873

